# Axonal Localization of Integrins in the CNS Is Neuronal Type and Age Dependent

**DOI:** 10.1523/ENEURO.0029-16.2016

**Published:** 2016-07-27

**Authors:** Melissa R. Andrews, Sara Soleman, Menghon Cheah, David A. Tumbarello, Matthew R. J. Mason, Elizabeth Moloney, Joost Verhaagen, Jean-Charles Bensadoun, Bernard Schneider, Patrick Aebischer, James W. Fawcett

**Affiliations:** 1 School of Medicine, University of St Andrews, North Haugh, St Andrews, KY16 9TF, United Kingdom; 2 Department of Clinical Neurosciences, John van Geest Centre for Brain Repair, University of Cambridge, Cambridge, CB2 0PY, United Kingdom; 3 Biological Sciences, University of Southampton, Highfield Campus, Southampton, SO17 1BJ, United Kingdom; 4 Laboratory for Neuroregeneration, Netherlands Institute for Neuroscience, Royal Academy of Arts and Sciences, Meibergdreef 47, Amsterdam 1105BA, The Netherlands; 5 Department of Molecular and Cellular Neurobiology, Center for Neurogenomics and Cognition research, Vrije Universiteit Amsterdam, Amsterdam 1081HV, The Netherlands; 6 Neurodegenerative Disease Laboratory, Brain Mind Institute, School of Life Sciences, Ecole Polytechnique Fédérale de Lausanne (EPFL), 1015 Lausanne, Switzerland

**Keywords:** adeno-associated virus, axon initial segment, dorsal root ganglia, integrin, retinal ganglion cell, sensorimotor cortex

## Abstract

The regenerative ability of CNS axons decreases with age, however, this ability remains largely intact in PNS axons throughout adulthood. These differences are likely to correspond with age-related silencing of proteins necessary for axon growth and elongation. In previous studies, it has been shown that reintroduction of the α9 integrin subunit (tenascin-C receptor, α9) that is downregulated in adult CNS can improve neurite outgrowth and sensory axon regeneration after a dorsal rhizotomy or a dorsal column crush spinal cord lesion. In the current study, we demonstrate that virally expressed integrins (α9, α6, or β1 integrin) in the adult rat sensorimotor cortex and adult red nucleus are excluded from axons following neuronal transduction. Attempts to stimulate transport by inclusion of a cervical spinal injury and thus an upregulation of extracellular matrix molecules at the lesion site, or cotransduction with its binding partner, β1 integrin, did not induce integrin localization within axons. In contrast, virally expressed α9 integrin in developing rat cortex (postnatal day 5 or 10) demonstrated clear localization of integrins in cortical axons revealed by the presence of integrin in the axons of the corpus callosum and internal capsule, as well as in the neuronal cell body. Furthermore, examination of dorsal root ganglia neurons and retinal ganglion cells demonstrated integrin localization both within peripheral nerve as well as dorsal root axons and within optic nerve axons, respectively. Together, our results suggest a differential ability for *in vivo* axonal transport of transmembrane proteins dependent on neuronal age and subtype.

## Graphical abstract

**Figure:**
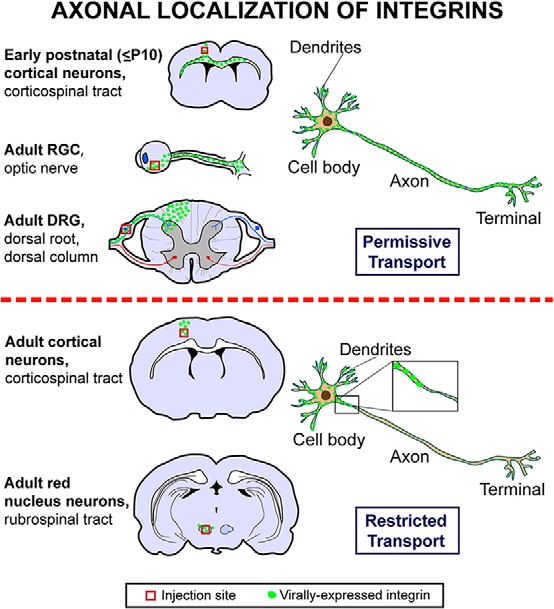

## Significance Statement

Most CNS neurons have an intrinsically low ability to regenerate their axons. This study has asked whether the transport into axons of integrins, the receptors that mediate growth through extracellular matrix, might reveal reasons for the poor regenerative ability of CNS axons. Tagged integrins were expressed in sensory, retinal ganglion cell, cortical, and red nucleus neurons. The integrins were transported down the axons of sensory and retinal ganglion cell axons, but not down the axons of adult cortical or red nucleus neurons. However, during the postnatal period of corticospinal axon growth, cortical neurons admitted integrins into their axons. The findings suggest that exclusion of integrins and other receptors from CNS axons may be a cause for their poor regenerative ability.

## Introduction

Deficiencies in the regeneration of adult CNS axons are due to several factors including inhibitory molecules in the lesion environment and a neuronal regenerative response that diminishes with neuronal maturation. Previously, integrin biology has been studied as a window into mechanisms of axon regeneration and their failure with maturation (Andrews et al., 2009; Eva et al., 2010, 2012; Tan et al., 2011; Franssen et al., 2015). Integrins are heterodimeric transmembrane proteins that bind extracellular matrix (ECM) molecules to induce cell proliferation, survival, and promote outgrowth (Hynes, 2002). These proteins are an integral part of CNS development, but many become downregulated in neurons upon CNS maturation (Hammarberg et al., 2000; Andrews et al., 2009). Consequently, when either integrins or their ligands are downregulated, axon growth fails. Specifically, when adult dorsal root ganglia (DRG) neurons are grown on marginal ECM substrates such as low levels of laminin and fibronectin, neurite outgrowth requires forced expression of α1 integrin and α5 integrin, respectively (Condic, 2001). It is therefore of interest that α9β1, the integrin that recognizes tenascin-C, the main extracellular matrix glycoprotein of the adult CNS, is developmentally downregulated. Following CNS injury, there is a substantial upregulation of tenascin-C without a concurrent upregulation of α9 integrin (Zhang et al., 1997; Tang et al., 2003; Andrews et al., 2009). As a strategy to induce axon regeneration in the CNS, this integrin has been re-expressed in the CNS. This allows sensory axons to grow prolifically on tenascin-C *in vitro* but addition of inhibitory substrates such as CSPGs and Nogo, as occurs *in vivo*, blocks this growth through inactivation of integrins (Tan et al., 2011). Forced activation of integrins can allow axons to overcome this inhibition (Hu and Strittmatter, 2008; Tan et al. 2011). By increasing the pool of α9 integrin in DRG neurons using adeno-associated virus (AAV), a modest increase in the regenerative response at the site of injury after dorsal rhizotomy and after a dorsal column spinal cord lesion has been demonstrated (Andrews et al., 2009). In addition, expression of the integrin activator kindlin-1 in DRG neurons only promotes limited regeneration in the spinal cord, whereas coexpression of α9 and kindlin promotes extensive regeneration and robust functional recovery (Tan et al., 2011; Cheah et al., 2016). An obvious next step is to use this combination to promote regeneration of intrinsic CNS axons. However, *in vitro* studies suggest that this strategy may fail because of the exclusion of integrins from CNS axons as they mature (Franssen et al., 2015).

Clearly, integrins can only stimulate axon regeneration if they are present in the axon at the site of damage. In the current study, we have asked whether integrins are transported into sensory axons including DRG and retinal ganglia neurons (RGCs), and into several types of adult neurons including adult cortical neurons, rubrospinal neurons, and we also evaluated early postnatal cortical neurons during their growth phase. This study has been enabled by improvements in viral technology that have made it possible to produce AAV vectors that can accommodate epitope-tagged full-length integrins. We show here that the presence in axons of virally expressed integrins *in vivo* is dependent upon neuronal type as well as age. Integrins are restricted to the cell body and dendrites of adult cortical and rubrospinal neurons whereas during the developmental growth period, we observed integrins in the axons of early postnatal cortical neurons. However in adult RGC and DRG neurons, which have better regenerative ability especially following conditioning lesions, integrins were present along the length of the axons. These results suggest that in two important pathways involved in motor control known to be incapable of axonal repair, integrins become excluded from the axons as they mature.

## Materials and Methods

### Integrin viral constructs

cDNAs encoding wild-type α9 integrin were obtained from Dean Sheppard (University of California San Francisco) as previously described (Andrews et al., 2009). cDNAs encoding wild-type α6 and β1 integrins and eGFP-N1 vectors were obtained from Charles ffrench-Constant (University of Edinburgh, UK) and cDNAs encoding β1 integrin-GFP were obtained from Martin Humphries (University of Manchester, UK). pcDNA3.1/V5-His was purchased from Invitrogen. cDNA of α6 and α9 integrin was cloned into eYFP-N1 using HindIII and KpnI restriction sites. V5 was PCR amplified adding on the AgeI and NotI restriction sites before being cloned into the a9integrin-eYFP plasmid upon removal of eYFP. Integrin lentiviral plasmids were constructed by PCR amplifying BclI and SacII onto the α6integrin-eYFP, α9integrin-eYFP, and β1integrin constructs, before cloning into the LV-PGK vector (Bensadoun et al., 2003). Integrin AAV plasmids using a short CAG promoter or a CMV promoter were constructed by inserting the tagged integrin constructs between the ITRs of AAV2 followed by a short poly-adenylation signal (49bp).

### Generation of viruses

#### Lentiviruses

The production, generation, and titration of lentiviral particles for LV-PGK-α9-eYFP, LV-PGK-α6-eYFP, LV-PGK-β1integrin, and LV-PGK-eGFP was performed with a four-plasmid system as previously described (Bensadoun et al., 2003).

#### Adeno-associated viruses

The production and titration of AAV serotype 2 and 5 vector particles for AAV5-CAG-α9integrin-V5, AAV5-CMV-α9integrin-eYFP, AAV2-CAG-α9integrin-V5, and AAV5-β1integrin-eGFP was performed as previously described (Fagoe et al., 2015). Briefly, a typical batch of AAV was produced in six 15 cm Petri dishes of HEK293T cells cultured in DMEM) containing 10% fetal calf serum and 1% penicillin/streptomycin. Transfer and helper plasmids were mixed in a ratio of 1:3 and cotransfected using polyethylenimine (linear MW 250,000, Polysciences). Three days post-transfection cells were harvested in Dulbecco's PBS (Invitrogen) and lysed by three freeze-thaw cycles. Genomic DNA was digested by adding DNAse I (Roche Diagnostics) and the AAV vector particles were purified from the crude lysate by the iodixanol gradient method (Hermens et al 1999; Zolotukhin et al 1999) and concentrated using an Amicon 100 kDa Ultra-15 device (Millipore). Concentrated AAV stocks were stored at −80^o^C until use. Titers (genomic copies/ml) were determined by quantitative PCR of viral DNA using primers against the CMV-enhancer sequence (Table 1).

**Table 1.** List of viruses produced and used for procedures

**Virus**	**Area injected**	**Serotype if AAV**
LV-PGK-α9integrin-eYFP	Cortex	—
LV-PGK-α6integrin-eYFP	Cortex	—
LV-PGK-eGFP	Cortex	—
LV-PGK-β1integrin	Cortex	—
AAV-CMV-α9integrin-eYFP	DRG	5
AAV-CAG-α9integrin-V5	Retina	2
AAV-CAG-α9integrin-V5	Cortex, DRG	5
AAV-CAG-β1integrin-GFP	Cortex	5

### Surgeries

Experiments were conducted in accordance with the UK Animals (Scientific Procedure) Act, 1986. Adult male Sprague-Dawley rats (250–400 g) were used for all cortical injections (adult and neonate), red nucleus injections, and intravitreal injections whereas adult male Lewis rats (250–400 g) were used for all DRG injections (Charles River Laboratories) (Table 2). Food and water were provided *ad libitum* and there was 12 h light/dark exposure. During surgery, adult rats were anesthetized in 1–2% isoflurane, in 2 L/min oxygen.

**Table 2:** List of animal groups with viral type used

**Area of injection and age**	**Virus**	**No. of animals**
SMC – Neonate	LV-PGK-α9integrin-eYFP	*n*=9
SMC – Neonate	LV-PGK-eGFP	*n*=10
SMC – Adult	LV-PGK-α9integrin-eYFP	*n*=3
SMC – Adult	LV-PGK-α6integrin-eYFP	*n*=3
SMC – Adult	LV-PGK-eGFP	*n*=5
SMC – Adult	LV-PGK-α9-eYFP +LV-PGK-β1	*n*=9
SMC – Adult	AAV5-CAG-α9integrin-V5	*n*=5
SMC – Adult	AAV5-CMV-β1integrin-GFP	*n*=6
SMC – Adult	AAV5-CAG-α9-V5 + AAV5-CMV-β1-eGFP	*n*=10
SMC – Adult + SCI	LV-PGK-α9integrin-eYFP	*n*=3
SMC – Adult + SCI	LV-PGK-α6integrin-eYFP	*n*=6
SMC – Adult + SCI	LV-PGK-eGFP	*n*=4
Red Nucleus – Adult	LV-PGK-α6integrin-eYFP	*n*=3
Red Nucleus – Adult	LV-PGK-eGFP	*n*=3
Red Nucleus – Adult + SCI	LV-PGK-α9integrin-eYFP	*n*=3
Red Nucleus – Adult + SCI	LV-PGK-α6integrin-eYFP	*n*=6
Red Nucleus – Adult + SCI	LV-PGK-eGFP	*n*=5
DRG – Adult	AAV5-CAG-α9integrin-eYFP	*n*=8
DRG – Adult	AAV5-CAG-α9integrin-V5	*n*=6
Retina	AAV2-CAG-α9integrin-V5	*n*=10

#### Cortical and red nucleus injections: adult

Adult cortical injection groups (LV-PGK-α6integrin-eYFP, *n*=3; LV-PGK-α9integrin-eYFP, *n*=3; LV-PGK-eGFP, *n*=5; AAV5-CAG-α9integrin-V5, *n*=5; AAV-CAG-β1integrin-GFP, *n*=6) sustained a single injection of 1μl LV or AAV into the left forelimb sensorimotor cortex (SMC) at (AP 1.5 mm, ML 1.5 mm, DV −1.5mm; Krajacic et al., 2009). Red nucleus injection groups (LV-PGK-α6integrin-eYFP, *n*=3; LV-PGK-α9integrin-eYFP, *n*=3; LV-PGK-eGFP, *n*=3) sustained a single injection of LV into the left red nucleus at (AP −5.9 mm, ML 0.7 mm, DV −7.0 mm). Injections were performed using a custom made 30 gauge stainless steel needle attached to a Hamilton syringe driven by an infusion syringe pump (World Precision Instruments) at 0.2 μl/min. One microliter was injected into the left SMC (LV or AAV) or into the left red nucleus (LV) over a 5 min period, followed by a 3 min period before needle withdrawal.

#### Cortical injection: neonates

Neonatal cortical injections (LV-PGK-α9integrin-eYFP, *n*=9; LV-PGK-eGFP, *n*=10) were conducted under hypothermic anesthesia with a single manual injection of 1 μl of LV into the developing left SMC at the following coordinates: ML 1.0 mm, AP −0.5 mm, DV −0.7 mm (Altman and Bayer, 1995), over the course of 1–2 min, followed by a 1 min period before needle withdrawal. Pups were returned to their mother immediately following the procedure. Half of each group was taken for immunohistochemical analysis at 5 or 10 d postinjection.

#### Dorsal root ganglia injection

DRG injections were performed as described previously with some variations (Andrews et al., 2009). Briefly, under anesthesia, a left hemilaminectomy was performed at cervical levels C5–C6 (AAV5-CMV-α9-eYFP, *n*=8) to unilaterally expose the C5–C6 DRG and dorsal roots or at lumbar levels L4–L5 (AAV5-CAG-α9-V5, *n*=8) to unilaterally expose the L4–L5 DRG and dorsal roots. One microliter injections were performed as described for the adult cortical injections except with a 33 gauge stainless steel needle attached to a Hamilton syringe driven by an infusion syringe pump at a rate of 0.1 μl/min, followed by a 3 min period before needle removal.

#### Spinal cord lesions

Cervical dorsal column crush lesions at C4–C5 were performed as previously described (Andrews et al., 2009) concurrent with the cortical injection. Briefly, under anesthesia (LV-PGK-α6integrin-eYFP, *n*=6; LV-PGK-α9integrin-eYFP, *n*=3; LV-PGK-eGFP, *n*=4), a laminectomy was performed at cervical levels C4 and C5 to expose the dorsal surface of the spinal cord. The dura was retracted and the dorsal columns, identified within the immediate extents of the dorsal root entry zones on either side, were crushed bilaterally for 10 s using finely-milled forceps at a depth of 2 mm. With regard to cervical lateral hemisection lesions at C4–C5 (LV-PGK-α6integrin-eYFP, *n*=6; LV-PGK-α9integrin-eYFP, *n*=3; LV-PGK-eGFP, *n*=5), access to the spinal cord was performed as described above. Following dura retraction and identification of dorsal roots, a scalpel blade was inserted into the spinal cord rostral to the root entry to a depth of 2mm with the cut extending laterally to the edge of the cord.

#### Intravitreal injections

Injections were performed as described previously (Bull et al., 2012). Briefly, under isoflurane anesthesia with local anesthestic applied topically to the cornea of the left eye, the left eyelid was gently retracted. Five microliters of AAV2-CAG-α9integrin-V5 (*n*=10 animals) was injected into the vitreous of the left eye using a using a custom made 30 gauge stainless steel needle attached to a Hamilton syringe. Care was taken during the injection to ensure the lens was not damaged. The needle remained in position for 1 min following injection before needle withdrawal.

### Immunohistochemistry

At the end of each experimental time point, animals were administered an overdose of sodium pentobarbital and transcardially perfused with PBS, pH 7.4, followed by 4% PFA, pH 7.4. Tissues (including brain, spinal cord, eyes, optic nerves, DRG, dorsal roots, and sciatic nerves depending on the paradigm) were removed and postfixed in 4% PFA and cryoprotected overnight in 30% sucrose in 0.1 m PB, pH 7.4.

#### Brain, spinal cord, dorsal roots, sciatic nerves, and ganglia

Brain tissue was sectioned coronally on a sliding microtome at a thickness of 40 μm and stored in PBS with 0.02% sodium azide at 4°C until further processing and analysis. Spinal cord tissue was embedded in OCT (RALamb UK) and sectioned longitudinally (spinal cord) or transversely with spinal cord attached (for dorsal roots, ganglia, and sciatic nerves) on a cryostat at 14 μm thickness, mounted on slides (Superfrost Plus, VWR International) and stored at −20°C until further processing and analysis.

Sections were washed with PBS and blocked in 10% NGS, 0.4% TX-100 in PBS. Primary antibodies, anti-β3 tubulin (1:400, mouse; Sigma-Aldrich), anti-GFP (1:500, rabbit; Molecular Probes), anti-V5 (1:250, mouse; Invitrogen), anti-GFAP (1:400, mouse; Sigma-Aldrich), anti-ankyrin G (1:100, mouse; Neuromabs), anti-ankyrin G (1:100, rabbit; Santa Cruz Biotechnology), anti-tenascin C (1:200, mouse; IBL America), anti-collagen IV (1:500, rabbit; Sigma-Aldrich), anti-fibronectin (1:100, rabbit; Sigma-Aldrich), and anti-laminin (1:500, rabbit; Sigma-Aldrich) were incubated overnight at 4°C. Sections were rinsed in triplicate in 0.1 m PBS and incubated with secondary antibodies (1:500, goat anti-mouse or rabbit AlexaFluor 568 or 488; Invitrogen), followed by a brief incubation with bisbenzimide (Sigma-Aldrich) nuclear stain. Slides were rinsed and coverslipped with Fluorosave. Certain antibodies (anti-tenascin C, anti-ankyrin G) required amplification using streptavidin. In these cases, pretreatment of the tissue with 0.3% H_2_O_2_ for 30 min was performed, along with incubation with biotinylated goat anti-rabbit or anti-mouse antibodies (1:500, Vector Laboratories) used in place of fluorescent secondary antibodies. Tissue was then incubated with streptavidin-conjugated to AlexaFluor 568 (1:250, Invitrogen).

#### Retinal whole mounts and optic nerve processing

Animals were perfused as described above and the eyes, optic nerve and brains were removed and postfixed overnight in 4% paraformaldehyde at 4°C. For retinal whole mounts, retinas from both eyes were dissected and prepared as flattened whole mounts by making four radial cuts (Bull et al., 2012). Retinal tissue was further postfixed overnight as above. Following PBS washes, tissue was incubated in blocking buffer (5% normal goat serum, 0.2% triton X-100 in PBS) prior to incubation overnight at 4°C in primary antibodies (rabbit anti-tubulin, 1:1000, Covance; mouse anti-V5, 1:250, Invitrogen) diluted in blocking buffer. Following further PBS washes, tissue was incubated overnight at 4°C in secondary antibodies (goat anti-mouse 488 and goat anti-rabbit 568, 1:750, Invitrogen) diluted in blocking buffer. Following the final PBS washes, retinal tissue was mounted on microscope slides and coverslipped with Fluorosave. For optic nerve sections, optic nerves were cryoprotected in 30% sucrose, embedded in OCT embedding media, cryosectioned longitudinally at a thickness of 14 μm and mounted onto Superfrost Plus microscope slides. Optic nerve sections were processed as described above for spinal cord cryosections using anti-V5 and anti-β3 tubulin antibodies.

### Microscopy and analysis

Fluorescence imaging was performed using a Leica DM6000 epifluorescent microscope, a Leica TCS SP2, and a Leica SP8 confocal microscope. Images were captured using LAS AF Leica software, and processed with Adobe Photoshop CS4.

## Results

### Axons are transported into the axons of developing postnatal cortical neurons

**Figure 1.**
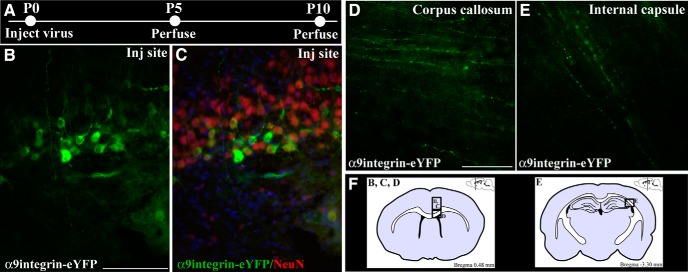
eYFP-tagged α9 integrin is transported into early postnatal cortical axons. Schematic of experimental design indicating the ages at which the lentivirus was injected and the time points of perfusion and tissue analysis (***A***). Fluorescent images of cortical injection site 5 d following LV-PGK- α9integrin-eYFP injection showing eYFP-labeled cortical neurons (green; ***B***) colabeled with NeuN (red) and bisbenzimide nuclear label (blue; ***C***). Fluorescent images of eYFP-labeled α9integrin within axons of the corpus callosum (***D***) or internal capsule (***E***), 5 or 10 d following cortical injection, respectively. ***F***, Coronal brain illustrations (left) indicate approximate area of injection site (of boxed area for B, C) and area of image of corpus callosum (in D) and (right) indicate approximate area of image of internal capsule (in E). Scale bars: ***B***, ***C***, 100 μm; ***D***, ***E***, 50 μm.

Integrins are vital for CNS development including neuronal migration and axonal elongation (Graus-Porta et al., 2001; Denda and Reichardt, 2007), so it would be expected that they would be transported to advancing axonal growth cones. We therefore examined axonal localization of integrins during the immediate postnatal period when certain classes of axons such as the corticospinal tract are still undergoing development and elongation. Initially, we used many integrin antibodies, but were unable to find one that demonstrated sufficiently good staining of endogenous integrins in tissue sections to show unequivocally whether or not integrins are present in classes of CNS axons. We therefore transduced neurons with tagged integrins that have been used previously in *in vitro* transport studies and which traffic normally in cellular vesicles to and from the cell surface (Eva et al. 2010, 2012; Franssen et al. 2015). Lentiviruses with their large cloning capacity (∼8 kB) were used in this study because they allow for expression of a fluorescently tagged, full-length integrin and have been shown to successfully transduce cortical neurons *in vivo* achieving maximum expression levels as early as 5–10 d (Hutson et al., 2012). In this group, we injected a lentivirus encoding eYFP-tagged α9 integrin under the control of a phosphoglycerate kinase promoter (LV-PGK-α9-eYFP) into the sensorimotor cortex of rat pups on the day of birth (P0) and assessed axonal localization 5 or 10 d later (P5 and P10; Fig. 1*A*). These early time points were selected based on minimal time required for *in vivo* lentiviral transduction and gene expression and the time window during which corticospinal tract (CST) axonal elongation is still ongoing. Results from this group demonstrated high levels of neuronal transduction illustrated by colabeling of NeuN and eYFP at the injection site, as early as 5 d postinjection (Fig. 1*B*,*C*). Quite strikingly at P5, we also observed eYFP-tagged α9 integrin localized in axons within the corpus callosum (Fig. 1*D*), whereas at P10 we observed YFP-tagged α9 integrin within axons of the internal capsule (Fig. 1*E*). In both cases, the localization was characterized by a punctate and vesicular appearance of the eYFP-tagged integrin (Fig. 1*D*,*E*).

### Adult DRG neurons transport integrins into both peripheral and central axons

**Figure 2.**
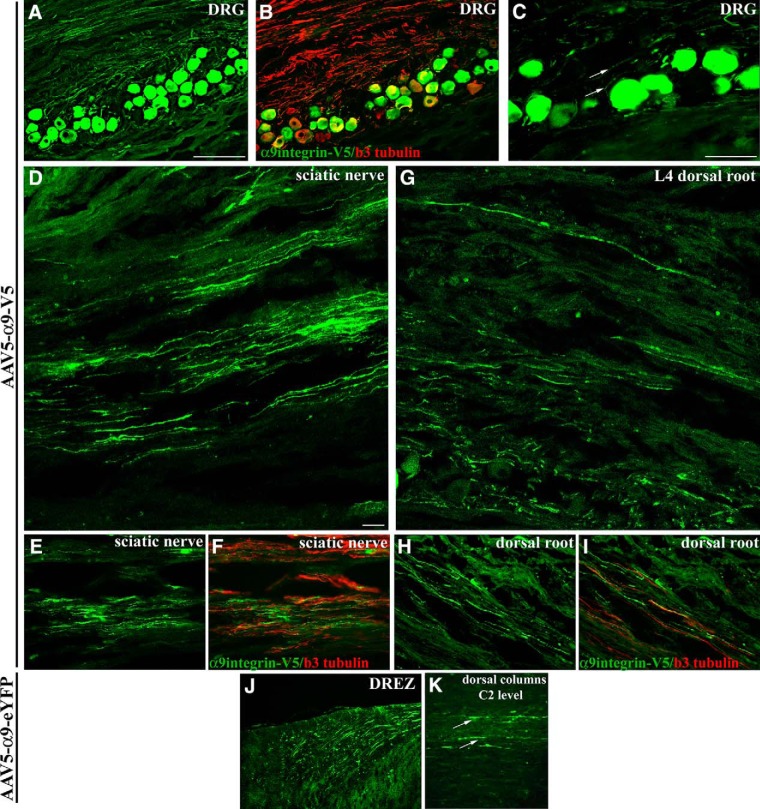
V5 and eYFP-tagged α9 integrin expressed in DRG neurons is transported to the central and peripheral branches of DRG axons. DRG neurons express α9 integrin-V5 (green in all panels) 4 weeks following injection of AAV5-CAG-α9integrin-V5 into the L4 and L5 DRG (***A***) including within the proximal neuronal processes (***C***, arrows), shown colabeled with β3 tubulin (red) (***B***). Confocal (***D***) and epifluorescent (***E***) images show V5-labeled α9 integrin within axons in the sciatic nerve, 4 weeks following DRG injection, colabeled with anti-β3 tubulin (red; ***F***). Confocal (***G***) and epifluorescent (***H***) images show V5-labeled α9 integrin within axons in the dorsal root, 4 weeks following DRG injection, colabeled with anti-β3 tubulin (red) (***I***). Epifluorescent image of eYFP-labeled α9 integrin (***J***, ***K***, green) in the axons of the dorsal root entry zone leading into the dorsal column, 6 weeks following DRG injection of AAV-CMV-α9integrin-eYFP into the C5 and C6 DRG (***J***), and in axons in the dorsal columns (***K***, arrows) observed in sagittal section at level C2 (***K***). Scale bars: ***A***, ***B***, ***J***, ***K***, 200 μm; ***C***, ***E***, ***F***, ***H***, ***I***, 100 μm; ***D***, ***G***, 20 μm.

We next investigated the presence of tagged α9 integrin in the axons of adult DRG neurons. For these experiments, we used AAV serotype 5 to achieve optimal transduction of DRG neurons because lentivirus will not transduce DRG neurons *in vivo* (Mason et al., 2010). Recently modified AAV plasmids (including plasmids with deleted WPRE sequences and small polyA sequences) made it possible to clone full-length α9 integrin fused to either an eYFP or V5 epitope tag. Cervical or lumbar DRG were injected with either AAV5-CMV-α9-eYFP or AAV5-CAG-α9-V5, respectively, and analyzed 3–6 weeks postinjection. We saw a high level of integrin within the cell bodies and in the axons within the DRG (Fig. 2*A*–*C*). After injection into the L4 and L5 lumbar DRG, examination of the sciatic nerve revealed clear localization of V5-tagged α9 integrin within peripheral axons (Fig. 2*D*–*F*). Likewise, examination of the central branch of the DRG, the dorsal root, revealed V5-tagged α9 integrin localized within these axons extending toward the spinal cord (Fig. 2*G*–*I*). Within the dorsal root entry zone, eYFP-tagged α9 integrin could be observed having reached and entered the spinal cord extending along the dorsal columns following injection into the C5 and C6 DRG (Fig. 2*J*,*K*).

### Adult optic nerve axons contain integrins

**Figure 3.**
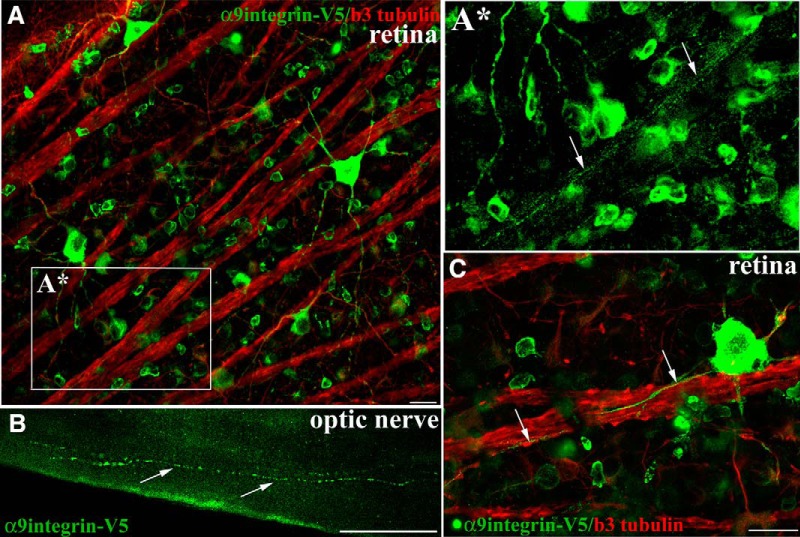
α9integrin-V5 expressed in adult RGCs is transported into optic nerve axons. Confocal images of flat mount retina show RGCs immunolabeled with anti-V5 (green) and colabeled with anti-β3 tubulin (red) 3 weeks after intravitreal injection of AAV2-CAG-α9integrin-V5 (***A***, ***C***). A*, A high magnification image (from ***A***) of integrin-containing axons in a fascicle (arrows) travelling toward the optic nerve. Epifluorescent images in ***B*** of optic nerve indicate V5-labeled α9integrin within axon fibers of the optic nerve 3 weeks following AAV injection. Arrows in ***C*** indicate V5-labeled axons following along the course of β3 tubulin axons. Scale bars: ***A***, ***C***, 20 μm; ***B***, 50 μm.

RGCs share some similarities with DRG in their response to injury and their regenerative ability, specifically with regard to the conditioning lesion response, including the significantly enhanced growth response following a peripheral preconditioning lesion (Leon et al., 2000). They also both respond to treatment with cyclic AMP (cAMP; Monsul et al., 2004). We therefore asked whether adult RGCs would transport integrin into optic nerve axons similar to DRG. In these experiments, intravitreal injections in adult rat were performed unilaterally using a serotype 2 AAV (AAV2-CAG-α9-V5), which is the optimal AAV serotype for transducing RGCs (Hellström et al., 2009). Three weeks postinjection we observed transduced cell bodies of retinal ganglia neurons along with axonal fibers colocalizing α9 integrin and β3 tubulin within axons extending toward the optic disc, as well as transport into the branching dendrites (Fig. 3*A*,*C*). Within the retina, we were able to see integrin transported into the axons of all the brightly transduced RGCs. Further examination into the optic nerve 3 weeks postinjection revealed the presence of α9 integrin-V5 as punctate vesicles throughout the length of some axons (Fig. 3*B*) including the presence of integrin in axons up to the chiasma resulting from this relatively short-term (3 weeks) experiment.

### Virally expressed integrin does not enter the axons of adult cortical neurons or adult red nucleus neurons

**Figure 4.**
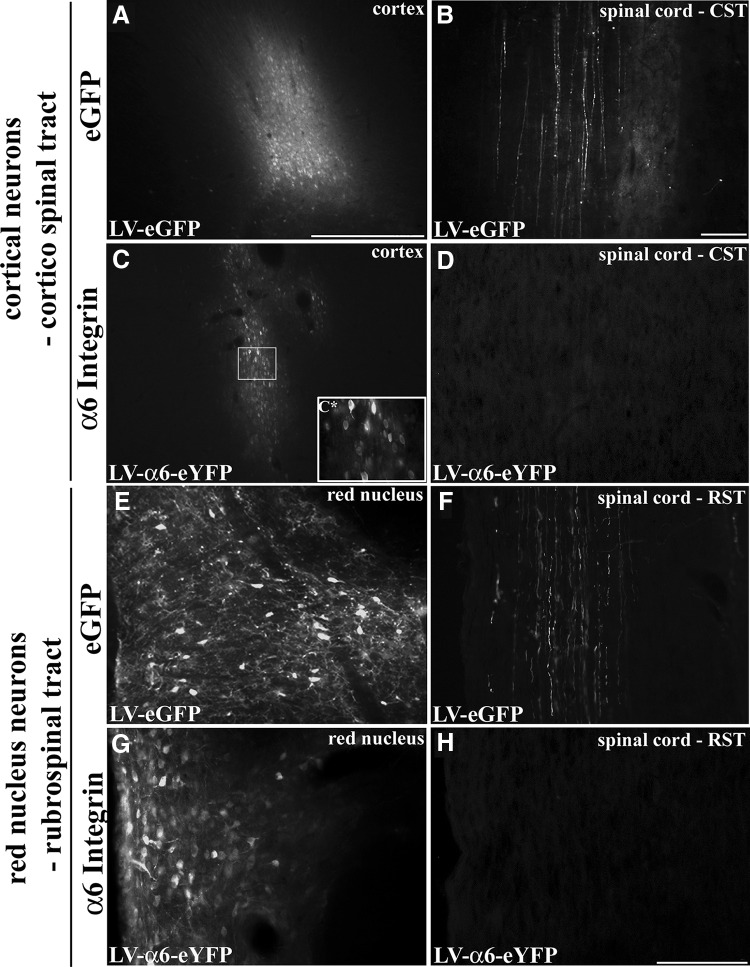
α6Integrin expressed in adult cortical or rubrospinal neurons is not transported down CST or RST axons. Adult motor cortex 3 weeks following injection of LV-PGK-eGFP (***A***) or LV-PGK-α6integrin-eYFP (***C***). In cervical spinal cord, axons are filled with eGFP in the CST following LV-PGK-eGFP cortical injection (***B***), but no integrins are observed after LV-PGK-α6integrin-eYFP injection (***D***). C*, High magnification view of LV-α6 integrin transduced cortical neurons. Adult red nucleus 3 weeks following injection of LV-PGK-eGFP (***E***) or LV-PGK-α6integrin-eYFP (***G***). Within the cervical spinal cord, only in the LV-PGK-eGFP injected groups are RST fibers found labeled with GFP (***F***) and not following LV-PGK-α6integrin-eYFP injection (***H***). Scale bars: ***A***, ***C***, ***E***, ***G***, 500 μm; ***B***, ***D***, 100 μm; ***F***, ***H***, 200 μm.

**Figure 5.**
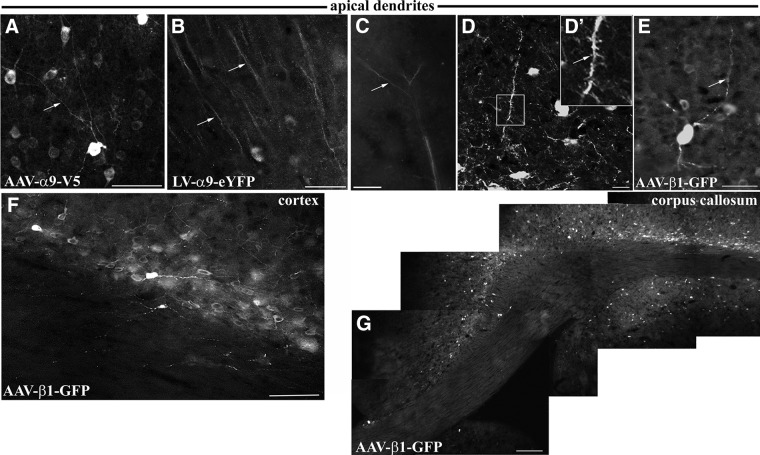
α9Integrin- and β1integrin-transduced adult cortical neurons express integrins in their dendrites but not in their axons. α9- and β1-transduced neurons (***A***–***E***) show prominent apical and basal dendrites (white arrows), but integrins have not entered the axon beyond the very proximal processes (***A***, ***E***, ***F***). ***B***, A region of cortex above the injection site demonstrating α9-transduced neurons with YFP-immunopositive integrin within apical dendrites. ***C***, Detail of YFP-immunopositive α9integrin dendritic arbors branching (white arrows) near the surface of the cortex. ***D***, A confocal image of YFP-immunopositive α9integrin within a dendrite with prominent dendritic spines (white arrows). ***F***, ***G***, The base of the cortex and the underlying white matter 4 weeks following AAV5-β1-GFP cortical injections demonstrating transduction of neurons throughout a wide area of cortex. Most of the white matter is devoid of tagged integrin, but a few fine processes of neurons very close to the white matter can be seen, demonstrating that labeled processes in white matter can be seen if present. ***G***, A composite showing subcortical white matter from the midline (left of picture) to lateral cortex. Although there are many integrin-transduced neurons in the overlying cortex, no integrin-containing axons are observed in the white matter of the corpus callosum. Scale bars: ***A***, ***C***, ***E***, ***F***, 50 μm; ***B***, ***G***, 100 μm; ***D***, 10 μm.

**Figure 6.**
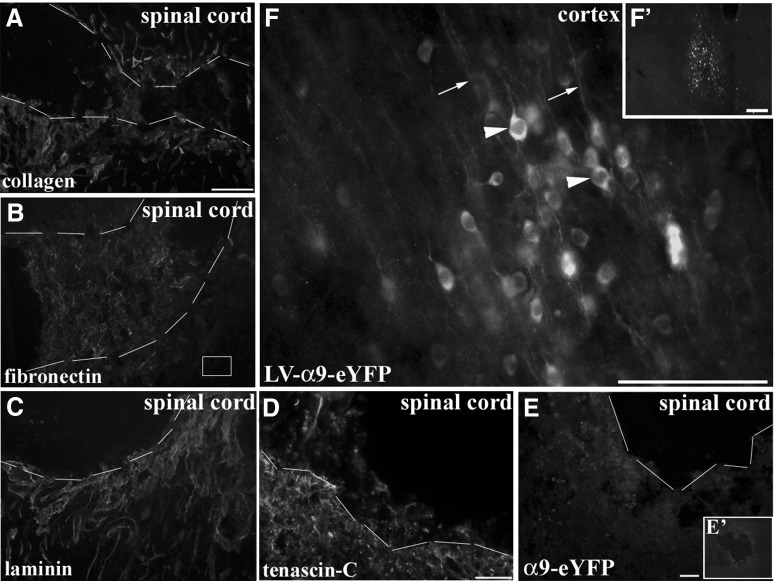
CNS injury induces upregulation of ECM expression but does not induce integrin localization in adult CST axons. Cervical dorsal column crush lesion leads to upregulation of ECM molecules such as collagen (***A***), fibronectin (***B***), laminin (***C***), and tenascin-C (***D***). Dashed lines in ***A***–***E*** indicate approximate borders of lesion site. Following injections of LV-α9integrin-eYFP (***E′***) into adult sensorimotor cortex with concurrent cervical spinal cord crush lesion did not induce CST axonal localization 3 or 6 weeks following injury and injection (***E***). High magnification image (***F***) demonstrates perinuclear appearance of neuronally expressed α9 integrin (arrowheads) also localized within dendrites (arrows). Scale bars: (in ***A***) ***A***–***F***, 100 μm; ***F′***, 200 μm.

To further assess axonal localization of virally expressed integrins in the CNS, we initially used lentivirus for *in vivo* neuronal transduction of adult cortical neurons. Unilateral injections into adult rat sensorimotor cortex resulted in a region of transduced neurons with either LV-PGK-α6-eYFP (Fig. 4*C*) or LV-PGK-α9-eYFP (Fig. 6*F*). This was comparable to the transduction efficiency found with injections of LV-PGK-eGFP (Fig. 4*A*). Upon examination of the cervical spinal cord, many GFP-filled corticospinal axons were visible in the LV-eGFP group (Fig. 4*B*). On the other hand, there was no indication of eYFP-tagged integrin in corticospinal axons in the cervical spinal cord expressing either α6 (Fig. 4*D*) or α9 integrin (data not shown). Assessment of the corticospinal tract more proximal to the injection site revealed no tagged integrin at any point. At the injection site, eYFP-tagged integrin (α9 integrin) was present in the cell body, dendrites, and in some proximal axonal processes of the transduced cortical neurons (Fig. 5*B*–*D*). In these cases, α9 integrin-eYFP could be localized within apical dendrites extending toward the outer layers of the cortex (Fig. 5*C*), and in some cases the labeling could be seen in dendritic spines (Fig. 5*D*). We saw no integrin transport beyond the proximal processes of the neurons, including the subcortical white matter or anywhere further down the corticospinal tract.

To confirm that the lack of integrin transport into adult CST axons was not due to experimental parameters of viral type (LV), viral promoter (PGK), or the large eYFP tag (722 base pairs) attached to the integrins, we repeated these experimental groups with consideration of these parameters. In these cases, we used AAV with an alternative promoter, CAG, and the small V5 epitope tag (42 base pairs) fused to the integrin (AAV-CAG-α9-V5). For these experiments, AAV5 was used as it has been shown to successfully transduce cortical neurons (Hutson et al., 2012), as well as other neuronal subtypes (Mason et al., 2010). Following adult cortical injections using AAV5-CAG-α9-V5, a larger and more diffuse area of neuronal transduction at the injection site was observed compared with LV, however, an absence of axonal localization of integrins beyond the proximal process of the axon remained (Fig. 5*A*). To show that this selective transport pattern is not restricted to alpha integrins, we also injected AAV-CAG-β1-eGFP, an AAV vector encoding tagged β1 integrin, and showed the same localization with transport into dendrites but no transport down axons (Fig. 5*E*–*G*). Normal intracellular trafficking of integrins requires the presence of the αβ heterodimers in order to be correctly transported from the endoplasmic reticulum through the Golgi to the plasma membrane (Tiwari et al., 2011). It is currently unclear how much β1 integrin, the binding partner of α9 integrin and α6 integrin, is expressed in adult cortical neurons *in vivo* despite its presence being documented in hippocampal and cerebellar neurons (Pinkstaff et al., 1999), and in neurons of the red nucleus demonstrated by *in situ* hybridization (Plantman et al., 2005). To examine whether cotransduction of α9 integrin and β1 integrin had an effect on axonal localization, we introduced both subunits by performing combined injections into adult rat cortex. These were performed in two groups: (1) LV-PGK-α9-eYFP and LV-PGK-β1 (untagged), and (2) AAV5-CAG-α9-V5 and AAV5-CAG-β1-eGFP. We saw no change to the overall localization of the eYFP- or V5-tagged α9 integrin, with integrins remaining in the cell body and dendrites (data not shown). We conclude that, in agreement with our previous observations *in vitro* (Franssen et al., 2015) integrins in mature cortical neurons are excluded from axons, but transported into dendrites (Fig. 5*A*–*E*).

Investigation within another motor tract in the adult, the rubrospinal tract (RST), revealed a similar pattern. Following lentiviral injections into the red nucleus of adult rat there were many neurons expressing high levels of GFP from LV-eGFP and LV-integrin (α6) injections (Fig. 4*E*–*H*). Examination of the rubrospinal tract in the cervical spinal cord revealed GFP-filled axons in the LV-eGFP group (Fig. 4*F*), although there was no indication of any eYFP-tagged integrin localizing in rubrospinal axons (Fig. 4*H*).

One of the main requirements for integrins to induce intracellular signaling and downstream cellular processes such as neurite outgrowth is for ligand binding to occur with ECM molecules (Reichardt et al., 1989; Hynes, 2002). In the above experimental groups, cortical injections were performed in uninjured naïve adult animals in the absence of an injury-induced upregulation of ECM, thus, although tenascin-C is present in the uninjured adult CNS, it could be suggested that transport into axons did not occur due to a lack of an ECM-stimulus, or that integrin transport might only occur after an axotomy. To address this issue, we performed cortical injections of LV-PGK-α9-eYFP or LV-PGK-α6-eYFP along with a concurrent dorsal column crush spinal lesion at C4/C5 injuring the CST. Alternatively, we also performed red nucleus injections of LV-PGK-α9-eYFP with a concurrent lateral overhemisection lesion at C4/C5 injuring the RST. Following spinal cord lesions there is a large increase in ECM molecules including tenascin-C, the ligand for α9β1 integrin (Andrews et al., 2009), and other ECM molecules such as collagen, fibronectin, and laminin, a ligand for α6β1 integrin (Fig. 6*A*–*D*). These cervical lesions did not result in the presence at the injury site of α6 integrin or α9 integrin in CST axons (Fig. 6*E*) or of integrins in RST axons (data not shown). Instead in these cases, we observed a similar localization of integrins within the cell bodies and dendrites at the injection site (Fig. 6*F*). To rule out distance as a factor influencing integrin transport to the injury site, a further group with a nearby cortical stab injury was included, but these cases also showed no axonal localization to the injury site (data not shown).

**Figure 7.**
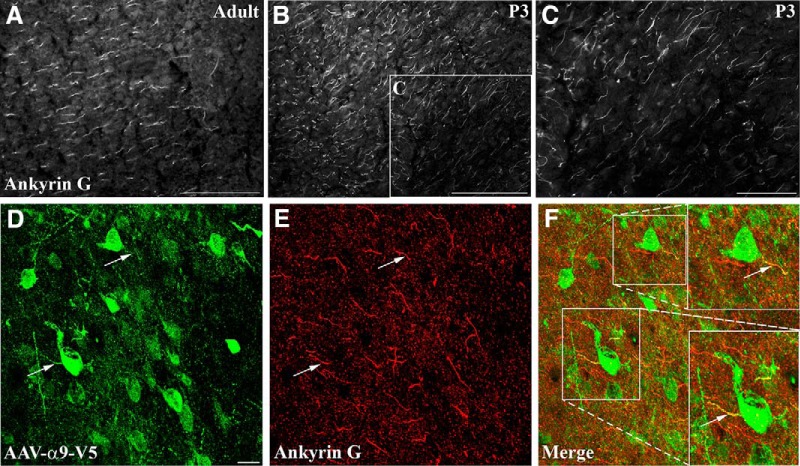
Ankyrin G is expressed in both early postnatal (P3) and adult cortical neurons, with integrin localization apparent in the axon initial segment in adult injection sites. Epifluorescent images of anti-ankyrin G immunolabeled cortex of adult (***A***) or P3 rat (***B***, ***C***). ***B***, Inset, Higher magnification in ***C***. ***D***–***F***, Confocal images near an adult cortical injection site (AAV5-CAG-α9-v5) with V5-immunopositive α9 integrin within neurons (***D***), colabeled with anti-ankyrin G (***E***), indicating that in some cases there was colocalization of virally-expressed integrin with the ankyrin G-immunopositive axon initial segment. Scale bars: ***A***, ***B***, 100 μm; ***C***, 50 μm; ***D***–***F***, 10μm.

There has been mounting evidence in the literature suggesting that the axon initial segment (AIS) acts as a selective barrier, only permitting some classes of molecules to access the axon (Song et al., 2009; Franssen et al., 2015). Although the role of the AIS in the localization of sodium channels is established, there was also some evidence in our *in vitro* study that the AIS might present a developmental barrier for molecules, such as integrins (Franssen et al., 2015; Normand and Rasband, 2015). Immunohistochemical analysis using antibodies against ankyrinG was performed on naïve early postnatal cortex (P3) and adult cortex. Results demonstrated that at both ages, ankyrinG-immunopositive structures were present in the axons of the cortex (Fig. 7*A*–*C*). Immunostaining for ankyrinG and α9 integrin-V5 in virally injected adult cortex showed that in many neurons integrins did not enter the initial segment, whereas in others there was some integrin in the proximal-most part of the axon, colocalizing with ankyrinG (Fig. 7*D*–*F*). In the cortex, therefore, the AIS is present at a time point when integrins are localized within axons (early postnatally) suggesting that ankyrinG by itself is not the barrier to axonal transport of integrins or equally that there is a developmental regulation of this barrier that occurs between early postnatal development and adulthood.

## Discussion

This study has focused on the axonal localization of virally expressed integrin receptors *in vivo*. The work was stimulated by demonstrations that expression of appropriate integrins can drive a growth response in damaged neurites and axons (Condic et al., 1999; Condic, 2001; Andrews et al., 2009). In particular, it has been shown that expression of the integrin α9, the receptor for tenascin-C, can enhance the intrinsic regenerative response of sensory axons in the spinal cord. Transduction of DRG with α9 integrin, particularly if accompanied by the integrin activator kindlin-1, enables cut axons to regenerate through the inhibitory tenascin-rich extracellular milieu of the damaged spinal cord (Andrews et al., 2009; Tan et al., 2012; Cheah et al., 2016). However, a recent *in vitro* study on cortical neurons indicated that integrins (both endogenous and expressed) are excluded from their axons as they mature (Franssen et al., 2015), suggesting that overexpression of integrin by itself will not stimulate regeneration of the axons of cortical neurons. In these CNS neurons, the mechanisms controlling transport into axons will also have to be addressed. In this study, we have addressed the issue of integrin distribution *in vivo.* We have expressed a variety of integrins in sensory neurons and three CNS neuronal types to see whether or not they are excluded from axons. We used viral-mediated expression of tagged integrins for two reasons. This is an established regeneration-inducing strategy and because integrin antibodies stain tissue sections poorly, potentially due to low endogenous expression levels in the adult CNS. We show that axonal localization of virally expressed integrins is highly dependent on neuronal type and age. Young cortical neurons during their growth phase as well as adult sensory and retinal ganglion cell neurons permit virally expressed integrins into their axons, whereas axons of adult motor (cortical and red nucleus) neurons do not contain integrins. Rather in the latter cases, integrins remain within the somatodendritic compartment, sometimes entering the very proximal axons where they coincide with the ankyrinG-immunopositive axon initial segment. Our finding in the rubrospinal tract is somewhat surprising due to published data that demonstrates that RST axons have been shown to regenerate for short distances into peripheral nerve grafts following certain treatments including BDNF (Kobayashi et al., 1997). These findings of integrin exclusion from the axon however, remain consistent regardless of viral type, viral promoter, integrin subunit, fluorescent/epitope tag, and axotomy. This confirms *in vivo* our previous *in vitro* findings that integrins are transported into the axons of immature cortical neurons, but are progressively excluded as the neurons mature (Franssen et al., 2015), that integrins are transported freely into adult DRG axons (Eva et al., 2010, 2012), and that integrins can be detected in adult retinal ganglion cell axons (Vecino et al., 2015).

The observation that integrin is transported into the axons of two populations of sensory neurons, adult DRG and RGC neurons, is interesting because both have a relatively high intrinsic regenerative ability. For example, following a (pre)conditioning lesion consisting of a peripheral nerve cut or crush (Richardson and Issa, 1984; Neumann and Woolf, 1999) or lens injury (Leon et al., 2000), both neuronal types have been found to have increased levels of GAP-43 (growth-associated protein, 43 kDa) and concurrently significant axon regeneration. Likewise, following neuronal application of cAMP, both axonal populations respond with increased growth after injury (Qiu et al., 2002; Monsul et al., 2004). Both neuronal populations continue to express integrins at higher levels than cortical neurons into adulthood (Vecino et al., 2015; Werner et al., 2000), so why do they not regenerate better in the CNS environment when cut? We suggest two reasons: first they do not express α9, the key integrin for interacting with the CNS extracellular matrix, and second any integrins present on their axons are inactivated by CSPGs and by NogoA (Hu and Strittmatter 2008; Tan et al., 2011). In support of these ideas, we have shown prolific regeneration of sensory axons in the spinal cord by transduction with α9 integrin and/or the integrin activator kindlin-1 (Andrews et al. 2009; Tan et al. 2012; Cheah et al., 2016). In the combined study applying both α9 integrin and kindlin-1, we have shown integrin transport into regenerating sensory axons in the dorsal column, and the fact that integrin-stimulated regenerating axons reached the medulla implies that active integrin was transported over this distance (Cheah et al., 2016).

Conversely, in our experiments with neurons in two motor pathways, the corticospinal and rubrospinal tracts, there was an obvious exclusion of integrins from axons. These pathways are well known to show only very low levels of regeneration following injury. Furthermore, neither a spinal cord injury-induced upregulation of ECM nor dual expression of both alpha and beta subunits were enough to stimulate transport in these axons. Likewise, in spinal cord lesions, transported proteins have been shown to accumulate at the terminals of cut axons (Ertürk et al., 2007), so had the integrins been axonally transported in adult CST axons postinjury, they should have accumulated and been present at the damaged axon ends even if less dense integrins within the length of the axons were below levels of detection. A correlation may exist between the endogenous regenerative capacity of axons and the ability of transmembrane receptors to localize/transport within axons. Moreover, it is not only integrins that are excluded from these axons, but also TrkB and IGFR (Hollis et al., 2009a,b). CNS axons appear to become specialized for connectivity through selective transport of presynaptic molecules and exclusion of growth-related molecules.

During the period of axon growth, integrins are important for enabling growth cone advance (Denda and Reichardt, 2007). It is therefore not surprising that we found that integrins are transported into corticospinal axons during their growth phase. At an early postnatal age, injection of LV-PGK-α9-eYFP in the sensorimotor cortex at postnatal day (P)0 resulted in α9 integrin localization in a punctate pattern within axons of the corpus callosum and the internal capsule at day P5 and P10, respectively, during which time axons are still elongating. It is also within this early postnatal age group that CST axon regeneration is possible following injury (Bates and Stelzner, 1993). The developmental change from integrin transport to exclusion correlates with a general age-related reduction in axonal transport that occurs within both CNS and PNS axons, which can be rescued in the PNS with a conditioning lesion (Milde et al., 2015).

For sensory neurons, a previous conditioning crush of the peripheral nerve can increase the regenerative ability of the central branch of the axons and stimulate local regeneration in the spinal cord (Neumann and Woolf, 1999). Retinal axons in the optic nerve show little regeneration unless a lens lesion or modification of signaling is applied (Leon et al. 2000; Sun et al. 2011). It will be interesting to see whether these various interventions alter integrin transport into axons. In the current work, lesion of the corticospinal tract made no difference to integrin transport into the axons. In the case of DRG neurons, we have obtained long-distance regeneration in the spinal cord of many axons with excellent sensory recovery through expression of α9 integrin and the integrin activator kindlin-1 in sensory neurons without needing to make a conditioning lesion (Cheah et al*.,* 2016).

Recent work has suggested mechanisms for axonal transport and selective exclusion of integrins. In sensory axons, integrins are transported in recycling vesicles marked by the GTPases Rab11 and Arf6 (Eva et al., 2010) and TrkB receptors in hippocampal neurons are associated with Rab11 (Huang et al., 2013). As cortical neurons mature, Arf6 transport of integrins becomes retrograde, acting to exclude integrins, driven by increases in the Arf6 GEFs, Efa6, and ARNO. Additionally, there are many studies that demonstrate that the axon initial segment acts as a filter at the axon hillock between the cell body and axon, allowing only select proteins to access the axonal compartment (Arnold, 2009; Song et al., 2009; Normand and Rasband, 2015; Eva et al., 2012; Petersen et al., 2014). However, our observation of the presence of an ankyrin G-immunoreactive axon initial segment as early as P3 in cortical neurons *in vivo* suggests that the AIS may exist but has not developed a full barrier function at an early age.

The findings in this study suggest differences in the transport of transmembrane integrin receptors and their subsequent localization within axons, which is dependent on neuronal subtype and age. In order for integrin receptors to induce a growth response, they have to be able to interact with the ECM of the external lesion environment and must therefore be transported to the tips of cut axons. In DRG and RGC axons this appears to be the case, and if transport into other CNS axons could be enabled, there is a strong potential for an enhanced regenerative response using integrin gene therapy.

## Synthesis

The decision was a result of the Reviewing Editor Michael Sofroniew and the peer reviewers coming together and discussing their recommendations until a consensus was reached. A fact-based synthesis statement explaining their decision and outlining what is needed to prepare a revision is listed below. The following reviewers agreed to reveal their identity: Andreas Faissner, Herb Geller

Reviewer #1

The manuscript makes the important point that integrins are not sorted into axons in a default mode. Rather, additional mechanisms are required. This is a novel and very important observation. Clearly, it opens the possibility that expression of a molecule as shown, for example, by transcriptome analysis, does not necessarily indicate its action in the axonal compartment and/or associated synapses.

I do not know why no visual abstract is included.

Specific comments:

Based on the observation that forced expression of Itga9 and kindlin stimulates regenerative growth of axons also in an otherwise inhibitory environment, the authors have examined the distribution of a9-integrin expressed in distinct neuronal classes via infection with Lenti- or adenoviruses. A variety of viral constructs was injected into distinct anatomical sub-regions of the rat brain. The resulting distribution of a9-integrins was followed and analysed in the respective neuronal populations. The salient finding of the study is that Itga9 does not distribute into certain types of CNS axons after infection. The authors suggest that this may be a reason for lack of regenerative capacity in territories enriched for ECM-ligands for that receptor. The paper is excellently written, the reasoning is clear and the results contain valuable information for the field of regenerative medicine. I have several comments or questions, as listed below:

1)The infection efficiency of retinal ganglion cells seems reasonable, yet not all neurons are labelled. Some fibres extending into the optic nerve are shown. Can the authors estimate which fraction of infected neurons in fact transports a9-integrin into the axonal compartment? While some neurons obviously do the efficiency of the strategy would require that nearly each infected neuron is able to transport the integrin into the axon.

2)Can the authors estimate the distances that can be reached by transfected itga9 in relation to the complete axon?

3)It is an intriguing observation that the a6- and a9-integrins are not transported into the spinal cord axons. The authors think that these integrins are distributed into dendrites of injected neurons. Have they evidence by staining for MAP2, a dendrite marker?

4)While ankyrin clearly seems expressed it would be of interest to see immunolabeling pictures for ankyrin in neurons infected with viruses to make the point that the tagged integrins are not localized to the ankyrin-labeld structures. This would support the conclusion that Itga6 and Itga9 are not sorted to ankyrin-positive structures to begin with, as proposed in this study.

5)In the experiments where both Itgb1 and a9Itg are expressed the co-expression in individual neurons in the double-injection cases is not really shown, as the Itgb1 is not tagged.

6)What is the quota of dying neurons in the injection experiments? Is the fraction different in the double-injection cases?

Reviewer #2

The authors demonstrate differences in integrin transport between different populations of neurons in vivo, which is interesting data.

Specific comments:

This manuscript represents a significant amount of careful work, with the main conclusion that there are major differences in the transport of integrins in different types of neurons. While the work is extensive, the interpretation that regenerative ability of neurons is tied to this integrin transport is still preliminary and would benefit from further experimentation.

Specifically, the authors suggest that RGCs and DRGs are more able to regenerate as compared to the other populations they studied, and tie this to integrin transport. However, these neuronal populations are only regenerative after conditioning lesions, yet the data here are taken with unconditioned lesions, where regeneration would not be expected. The images of RGC regeneration show lots of transfected cells in the retina, with only a low level of co-expression with tubulin, and very few labeled axons in the optic nerve, many fewer than would be needed for recovery of function. This is puzzling. Because regeneration of these populations is only found after preconditioning lesions, it would be important to present data on integrin transport in this situation.

The authors repeatedly argue that the alpha-9 receptor is responding to tenascin-C, so it would be important to show localization of tenascin-C along with the other ECM molecules, rather than data not shown.

Online discussion of reviews:

During the online discussion, there was agreement about the need to address all of the specific points raised by both reviewers.

In particular the reviewers discussed and agreed on the issue that DRG or RGC regeneration is seen only after conditioning lesions (or equivalent stimulations), that the other systems studied did not have equivalent known manipulations that are effective in increasing regenerative capacity, and that there was insufficient attention paid to how such manipulations in only the DRG or RGC systems would impact on the main observations of the paper. This was regarded as an important point that should be addressed, preferably with experimental data and at the very least with a thorough discussion.
